# Retrospect of the Medical Sciences

**Published:** 1843-07-22

**Authors:** 


### RETROSPECT OF THE MEDICAL SCIENCES.

### CASE OF STRANGULATED INTESTINES, FROM ROTATION OF THE SIGMOID FLEXURE—WITH REMARKS.

BY JACOB BIGELOW, M. D.

The Hon. Hugh S. Legare, Attorney-General of the United States, arrived in Boston on Friday, June 16th, and although fatigued by a hasty journey from Washington, was well enough to make calls on some friends in the evening. At 1 o'clock in the night he was seized with frequent abdominal pains, resembling those of colic, and called Dr. Thomas, of Washington, then lodging in the same hotel, to his assistance. During the remainder of the night, and the whole of the next day and night, he was affected with pains alternating with intervals of ease, without constitutional disturbance, and agreeing in character with those of previous attacks to which he had been liable for more than two years, the last occurring in March preceding. Various laxatives and enemata were resorted to, togather with counter-irritants, but without removal of the constipation and pain.

Early on Sunday morning I was called to meet Dr. Thomas at Mr. L.'s lodgings at the Tremont House. I found him then suffering frequent paroxysms of pain, which he referred mostly to the lower abdomen, without distinction of side, but which sometimes mounted above the umbilicus. The pulse was at this time 60, the skin natural, with no tenderness on deep pressure of the abdomen in any part, no meteorism, no nausea. Opiates and other remedies were proposed to him, but declined, on the ground that laxatives and mechanical means had relieved his former attacks. During the morning two doses of Epsom salt, with infusion of senna and tincture of hyoscyamus were given, with frequent enemata both aqueous and stimulating, without effect. The pains did not increase, but a troublesome degree of tenesmus made it necessary to suspend the enemata. Elastic tubes were passed throughout the rectum, and water injected through them in the manner recommended by Dr. O'Beirne, but they could not be carried into the sigmoid flexure. His strength meanwhile remained good and his general condition stationary.

At 6 P. M. he was removed without difficulty to the house of a friend, where he was immediately put into a warm bath of 106 deg., from which he expressed great relief and satisfaction. He was then put to bed, and 60 drops of laudanum were administered in two doses. In about an hour, the relief not being perfect, 40 drops of Munn's elixir of opium were given, soon after which he fell into a quiet sleep, and so remained for about three hours. Conditional directions were given for repeating the opiate, but it was not found necessary till near morning, when he took 20 drops of the elixir and slept an hour or two more. On Monday morning at 5 o'clock I found him more comfortable than before, skin temperate, pulse 64, abdomen not tender but beginning to be tympanitic. Castor oil and senna, with hyoscyamus, were now given and retained, and enemata, fomentations and sinapisms were resumed as before. The pain did not return with the same severity as before, but meteorism rapidly increased, with restlessness and tenderness on pressure. At 9, A. M., the pulse was 80, and before 12 it was 100. The face of things having become very serious, Dr. Thomas being absent from the city, I requested farther consultation, and Dr. Warren was called in. The abdomen was freely leeched and rubbed with croton oil. Various ineffectual attempts were made to overcome the obstruction of the intestines by the introduction of various tubes, by inflation of the rectum with a bellows, and by the tobacco injection administered twice. Under this last remedy he said he felt excited, was stronger but more agitated, and his pulse rose from 130 to 140, with increased force. Each injection contained half a drachm in infusion, and was retained nearly half an hour without narcotism or prostration. During the night the patient was restless, retaining his muscular strength in a considerable degree, and frequently getting up to the close stool in the belief of an approaching evacuation. There was never any vomiting nor nausea; the mind was clear, and the natural decisive tone of voice continued. He complained occasionally of a sense of burning at the epigastrium and upper abdomen. About half an hour before death he got up without assistance, and on lying down asked urgently for water. On receiving it, he pushed it away, saying it was filled with ants. A white paper was then shown him, to which he applied the same remark. On being told it was an illusion of sight, he put forth his hand for the glass, but missed it, said a few words incoherently, leaned back, and expired quietly at half past 5.

*Autopsy, seven hours after death.*—Externally the limbs were very rigid, and there was much lividity about the head and back. The abdomen was greatly distended. On laying it open the cavity seemed nearly filled by the sigmoid flexure of the large intestine, which extended across the abdomen into the right hypochondrium, and was in a state of such distension, that its external circumference was in one place fifteen inches. It had a dusky green colour, as if from commencing gangrene, but there seemed to be no softening, nor diminution of the natural polish. The two extremities of the flexure connected with the colon above, and rectum below, were felt to be twisted together about the mesentery as an axis, into a firm cord or neck, about an inch in diameter; and on being carefully untwisted, the whole included portion was found to have made four turns, or two entire revolutions upon itself. There was no line of demarcation between the healthy and strangulated portions, nor was there any appearance externally of old disease about this part. The small intestine and the colon were moderately distended, but the rectum was rather contracted. The cavity of the peritoneum contained a small quantity of turbid reddish fluid, and in one place there was recent lymph upon the small intestine, but there were no other appearances of inflammation. Owing to the state of the body and the place of examination, the intestine was not opened, and no farther dissection made.

*Remarks.*—Internal strangulation, we have reason to believe, is a fatal disease, except in rare instances in which a spontaneous restoration of the parts may under favourable circumstances have taken place. But the resources of art are for the most part unavailing, from our ignorance at the time of the nature and place of the lesion, and from the inaccessible situation of the part, unless by a dangerous operation, not to be justified under any diagnosis which can be seasonably made out. Among the various causes known to have occasioned strangulation, the rotation or twisting of the intestine is less common than some others. Yet in addition to the case which has now been described, two others have occurred in this city, under the observation of Drs. Homans and J. B. S. Jackson, the record of which I have seen, in which fatal strangulation occurred from the torsion or twisting of the sigmoid flexure.

Professor Rokitansky, of Vienna, in a work on internal strangulations of the intestines, divides these lesions into three species. Of these, the second species consists in the rotation of one part round an axis most commonly formed by some other part. It appears to be the result of his experience that rotation round the mesentery as an axis can happen only to the small intestine. But it appears from the case above detailed, and the two others above alluded to, that the large intestine is capable of undergoing this rotation, and from its anatomical position, no part seems more exposed to this change of situation than the sigmoid flexure.

From remarks made by Mr. Legare during his illness, it is believed that in some of his former attacks of colic and constipation, relief was obtained by the introduction of the elastic tube beyond the seat of the stricture. This happy result is to be ascribed to the spasmodic character of the obstruction then existing. But when the intestine is rendered impervious by mechanical strangulation, it is evident that an instrument would sooner perforate the coats of the canal, than admit of being forced through the closed and tortuous passage. In the present case, tubes, some of which were two feet in length, were introduced into the rectum, and water injected through them continually to facilitate their progress. But the more flexible tubes were bent into a coil in the rectum, and the more rigid ones were irrisistibly stopped at the sigmoid flexure, and could not be further forced without danger of perforating the intestine, an accident well known to have followed injudicious and violent efforts.—*Boston Med. and. Surg. Journ.*

---

CALUMBA root, in powder, has been found very serviceable in the removal of vomitings not dependent on any acute inflammation of the stomach and other viscera. In cases of spasmodic or other nervous irritation it is advantageously combined with an opiate. On the occurrence of acid risings with which persons of weak digestion are often troubled, magnesia is a suitable agent to combine with the calumba.—*London Lancet.*

---

### CONCISE HISTORICAL SKETCH OF THE PROGRESS OF PHARMACY IN GREAT BRITAIN.

BY JACOB BELL.

Pharmacy was originally in the hands of the physicians, who prepared their medicines themselves, or superintended the preparation of them. The science of medicine was then but little understood, and was often confounded with witchcraft; in fact the Greek word φαρμακεω signifies either to practise witchcraft or to use medicines.

The first act of parliament relating to the medical profession was passed in the year 1511; by this the faculty of medicine was vested in one body of practitioners who exercised medicine, surgery, and pharmacy. The physician's assistants were styled apothecaries, and these soon began to transact business on their own account.

In the year 1518 the College of Physicians was established. In 1540, their powers were increased, and they had the right of entering the houses of the apothecaries in London, examining the drugs, and destroying such as they deemed unfit for use. In the same year the barbers and surgeons were united into one company, but the surgeons were prohibited from shaving, and the barbers were restricted from performing surgical operations.

In 1695, the question was tried as to whether surgeons were entitled to administer medicines internally, when it was decided "that no surgeon, as a surgeon, might practise physic for any disease."

It is uncertain at what time the physicians gave up the practice of preparing their own medicines, but it appears that in 1617 the apothecaries obtained a charter constituting them a company by themselves wholly to be employed in the business of pharmacy. It was enacted at the same time that no grocer should keep an apothecary's shop, and that no surgeon should sell medicines.

The first Pharmacopœia was published by the College of Physicians in the year 1618. This was the first step towards the improvements which have since obtained in pharmacy. This was, however, a very imperfect production, the compounds chiefly employed being either empirical nostrums, or mixtures of great numbers of substances, without any reference to scientific principles. Thus the Mithris was a compound of seventy-two ingredients, and many others were equally as absurd.

Culpeper, in his translation of the Pharmacopœia, gives a list of the remedies derived from the animal kingdom, contained in the Pharmacopœia. The following will serve as a specimen, with Culpeper's remarks in parentheses.

"The fat, grease, or suet of a duck, goose, ell, bore, heron, thymallos, (*if you know where to get it,*) dog, capon, beaver, wild-cat, stork, hedge-hog, hen, man, lyon, hare, kite, or jack, (*if they have any fat I am persuaded 'tis worth twelvepence the grain,*) wolf, mouse of the mountain, (*if you can catch one,*) . . . . . . album græcum, east and west bezoar, stone taken out of a man's bladder, viper's flesh, the brain of hares and sparrows, the rennet of a lamb, kid, hare, and a calf and horse too. (*They should have put the rennet of an ass to make medicine for their addle brains.*) The excrement of a goose, of a dog, of a goat, of pigeons, of a stone horse, of swallows, of men, of women, of mice," &c. &c.

But though Culpeper justly ridicules the College of Physicians for inserting such wretched trash, some of his own remedies are by no means first-rate, thus he says—"The head of a cole-black cat being burnt to ashes in a new pot, and some of the ashes blown into the eye every day, helps such as have a skin growing over their sight."

About this time chemists begin to be mentioned as a class of men who prepared, for the use of the apothecaries, the "chemical medicines," or those o[illegible] mineral origin, and which were subjected to fire.

In the year 1671, however, the Society of Apothecaries, which had continued to prosper, established [illegible] chemical laboratory, in addition to the dispensary thus uniting in one establishment the preparation [illegible] chemicals and galenicals. This institution was a[illegible] first on a small scale, but the superior quality of th[illegible] articles supplied, soon led to an application on th[illegible] part of others to participate the benefit. In a fe[illegible] years, consequently, from this time, the Compan[illegible] became a trading body, and supplied all cust[illegible] mers.

In 1694, apothecaries were exempted from servin[illegible] the offices of constables, scavengers, &c.; by th[illegible] time they had increased so rapidly in numbers, as [illegible] have become a very influential body. By practisi[illegible]

medicine as well as pharmacy, they excited the jealousy of the physicians, between whom and the apothecaries a violent contest now rose; pamphlets were published on both sides, and the dispute ran high, till at last the physicians resolved to put down the apothecaries by establishing dispensaries, where they supplied medicines on reasonable terms, made up under their own superintendence.

The dispensaries prospered, but in process of time, the physicians becoming weary of the drudgery of the business, established the assistants whom they had employed and instructed at these places, as dispensing chemists on their own account; some of the apothecaries, finding the business profitable, adopted the same course, and from this we may date the origin of chemists and druggists.

In 1723, the College of Physicians were again empowered to visit the shops of apothecaries, and examine the drugs; this right, however, was not always exercised in a very impartial manner, nor indeed does it appear that the persons employed were always very competent.

Thus, an anecdote is related, that some of the examiners coming to an apothecary's shop, found a pot with *ung. album* written on it: the gentlemen got about the pot and examined it; each gave his opinion—one said it was hard, another that it did not smell enough of camphor, a third that it ought to be malaxed with some oil, while a fourth proposed treating it in a summary manner by throwing it out of doors. In the height of their dispute, the boy coming in, entreated them to stay their hands, assuring them that it was perfectly good, but only put into the wrong vessel. "What is it then?" said one of the learned. "Why, gentlemen," said the boy, "'tis white dog's ——."

In the Pharmacopœia of 1746, a great improvement had taken place over that which preceded it, many of the complicated and absurd formulæ were removed, and considerable progress had been made in the chemical part. The Mithridatum with its forty ingredients, and Theriaca Andromachi with about sixty, were still however allowed to remain.

The origin of the Botanic Garden at Chelsea is involved in some obscurity, but it is supposed to have been founded prior to the year 1673. In 1722, Sir Hans Sloane, who had purchased the manor, granted it on lease to the Society of Apothecaries, on condition that they should present annually to the Royal Society fifty distinct plants, till the collection amounted to 2000, and also that the garden should be appropriated to the purpose of cultivating plants, instructing students, and advancing science.

Towards the latter end of the last century, disputes having arisen between the chemists and apothecaries, the latter formed themselves into a Society under the title of the General Pharmaceutical Association of Great Britain, for the purpose of preventing chemists from vending pharmaceutical preparations, compounding physician's prescriptions, &c. The result of their exertions, however, was not so successful as was anticipated, and the Society shortly broke up without having effected much against the druggists, whom they had threatened to destroy.

For the remainder of the account of the differences between the apothecaries and chemists and druggists, we must refer to the pamphlet itself, in which our readers will find much to instruct and amuse them.—*Medico-Chirurgical Review.*

PYELITIS.

Having in his former volumes disposed of the inflammations of the proper tissues of the kidneys themselves, M. Rayer proceeds in that now before us to examine the inflammatory affections of the structures more or less intimately connected with those organs. Inflammation of the pelvis and calices first engages his attention; and of this disease the author may be said (if it be allowable for a moment to adopt the usual but rather pompous phraseology of our neighbours under such circumstances) to have created the history. Pyelitis, the name proposed for the affection, (from πευλος, pelvis,) is in truth, in all that regards precise knowledge of its seat and anatomical characters, but more especially of its symptoms, essentially a novelty to the observer of renal disease. It was invariably confounded during life with actual nephritis, and is still so confounded by a majority of persons; while on the other hand it is not a little amusing to find certain writers, after having had their confusion of ideas on the subject cleared up by the lucid exposition of M. Rayer, suddenly discovering that this very pyelitis was among their oldest pathological acquaintances. That these writers, whose conscientiousness we by no means desire to gainsay, had seen cases of pyelitis before M. Rayer wrote is indubitable; but that they had acquired the power of distinguishing it from nephritis, that they had even suspected the existence of such a distinct and in many respects totally different affection, is what, until proof of the affirmative be produced, we conceive ourselves at liberty to doubt altogether.

*Pathology and Diagnosis.*—Pyelitis, occasionally occurring as an acute, is most commonly a chronic, disease; and may be considered to be of various species according as the character of the inflammation and the nature of the causes producing it vary; thus we have the simple, calculous, blennorrhagic, gangrenous species, &c. The disease may implicate the entire of both pelvis, or part only of one; it may even be confined to one or both calices.

The anatomical characters of that *acute* form of the affection may be stated as follows: General or partial injection of the mucous membrane, accompanied occasionally with minute petechiæ, or effusions of blood sufficiently considerable to deserve the name of blood in the cavity of the pelvis or calices, are among the more remarkable appearances. In some cases partial deposits of whitish or grayish pseudo-membranous matter are found adheriug more or less closely to the surface, a circumstance of importance, as contributing to show that the tendency to formation of plastic exudation, though rarely brought into action on mucous surfaces, yet exists in them all. When the accumulation of this matter is sufficiently abundant to impress a special character on the inflammation, when this is in fact *pseudo-membranous*, the orifices either of the calices, pelvis, or ureter may be blocked up and effectually obstructed; this is the only circumstance under which such obstruction occurs in the acute disease, as thickening of the mucous membrane itself is generally of slight amount. When caused by retention of urine, acute pyelitis may be attended with dilatation of the pelvis and calices and flattening of the mammillæ. The urine in those cases is invariably mingled with pus, discoverable with the microscope when otherwise invisible. The other physical and chemical properties of the fluid vary with the species of pyelitis; it may contain urates, uric acid, phosphates, albumen, &c.

In *chronic* pyelitis, the most common colour of the mucous membrane is dull white; varied here and there with reddish or more frequently brownish injection; if the disease be of very old standing, grayish or slate-coloured patches are far from uncommon appearances. The veins on the exterior of the kidney are sometime much enlarged, and so arranged as to form networks. The mucous membrane may have undergone thickening to such an amount as to entail contraction of the orifices of the calices, and even to cause an apparent conversion of those canals into fibrous tissue. We strongly doubt this conversion as described by M. Rayer, and apprehend that in such cases the mucous membrane has been originally destroyed by ulceration or otherwise, and exudation or plastic matter, thrown out on the new surface, hardened into the fibrous-like tissue he has figured. This is probably at least the most common case; there are others no doubt in which the exudation of similar matter *under* the mucous membrane leads to a like result as regards apparent thickening of the mucous membrane and obstruction of the channels for the passage of the urine.

A curious condition observed by M. Rayer in both forms of pyelitis, but in most rare cases, is an eruptive of transparent vesicles like the sudamina so common in the typhoid fever of Paris.

Ulcerations of various extent and depth add to the amount of organic change in many cases. They vary in size from a pin's head to that of a sixpence, or more, and may spread so deeply as to cause complete perforation of the pelvis and the establishment of fistula, communicating with the surrounding cellular membrane, the peritoneum, the duodenum, the large intestine, &c. It is important to remember that these perforations may occur quite independently of distension of the pelvis, although renal fistulæ do certainly occur most frequently as the final morbid change in cases of atrophy of the kidney, with distension of the pelvis into a huge multilocular cavity. These ulcerations, more especially when of moderate dimensions, are susceptible of cicatrization, and when so cured present precisely the anatomical varieties observed in healed ulcers of the stomach, and, we may add, of mucous membranes generally.

With the enormous tumours produced by distension of the pelvis with urine, pus, blood, &c., most persons are tolerably familiar, at least as regards the fact that tumours of this description, extending from Poupart's ligament below, to the upper limits of the abdomen above, and driving the liver or spleen upwards into the chest, are not very uncommon morbid appearances. The atrophy of the renal substances, which coadvances with the accumulation in the pelvis, until this is distended into a simple membranous sac, readily explains the error of the older observers, ascribing these changes all to ulceration and suppurative destruction of the kidney itself. There is an infinitely rarer species of atrophy of this organ, pointed out by M. Rayer, in which it retains its natural form, while gradually reduced to a less size than that of a new-born infant, the pelvic accumulation proceeding as in the ordinary cases.

Chronic pyelitis may be of cancerous or tuberculous origin. In the latter case M. Rayer, as Dr. Carswell had done previously, finds "tuberculous matter" adherent to the mucous surface. We shall not stop here, however strongly tempted, to argue the question of the absolutely tuberculous nature of this matter; for we could not give fair expression to our convictions on the subject, without entering into a disquisition upon the meaning of the word tubercle (or rather upon the nature of the matter of tubercle) in nowise compatible with the general subject now under consideration. We must venture, however, to question the justness of MM. Carswell and Rayer in terming this substance tuberculous, if by the word we are to understand matter of the same essential nature as the well known pulmonary product.—*Notice of Rayer's Treatise on Diseases of the Kidneys, in Brit. and For. Med. Rev.*

---

### UNIQUE SPECIES OF HERNIA.

*Death—Examination.*

MM. Carteron and Saussier, of Troyes, report the case of a woman, the mother of fifteen children, and who had, for about twelve years, had a crural hernia of the left side, very little perceptible externally, and which was usually without difficulty kept from protrusion by a truss. One day, during an effort at stool, the hernia suddenly protruded. It was returned without difficulty by the patient; but after a few minutes a plucking sensation began to be felt in the left iliac fossa, which gradually extended to the umbilical region, increasing in intensity until violent colic was experienced. The woman was obliged to lie on her back, immoveable, and with the legs and thighs flexed; the temperature of the body was below the healthy average; pulse weak, and beating only from 45 to 50 per minute; the abdomen inflated; vomiting and various other symptoms were present, indicating the presence of a strangulated hernia. We may pass over the details of the treatment; it was unsuccessful, and the patient died in less than two days.

On opening the body, about two quarts of blood were found effused, half into the cavity of the peritoneum, and half into that of the small intestine (*celle de l'intestin grèle!*). The stomach (duodenum) and upper part of the jejunum contained a small quantity of air; the lower fifth of the small intestine and the large intestine were entirely empty. At the junction of the lower fifth with the rest of the small intestine, a mass of the ileum, eight inches in length, with a portion of mesentery, was found strangulated within a recent slit torn in the parietes of a herniary sac to be presently described. No perforation appeared in the intestine, nor was that portion of the mesentery engaged at all diseased.

The herniary sac, which extended from close to the uterus down to the crural arch, was attached at its extremity by firm bands to the thigh-joint (*racine de la cuisse.*) It opened above by an oval orifice, capable of admitting the forefinger, a little more than an inch from the commencement at the uterus of the round ligament. It accompanied this ligament to near the internal inguinal ring, and then left it to pass under the crural arch, where it terminated in a cul-de-sac, capable of admitting a small egg. At a little distance from its orifice was a slit larger than the orifice itself, and opening from a supplementary sac (*laquelle termine un infundibulum, un appendice veritable,*) a subdivision, as it would seem, of the sac which may thus be considered double. This appendix, or supplementary sac, was about one inch and a half in length by a like breadth, and free in the abdomen. Its form was conical, its larger end being appended to the principal sac. The parieties of both sacs were formed by the peritoneal fold forming the broad ligament of the uterus. It was in passing from the principal into the smaller sac, and across this through the newly-formed slit, that the mass of small intestine and mesentery had become constricted. The mesentery had become involved at a point so near to its attachments to the vertebral column that by the attempt to straighten the body during life a considerable strain was exercised on the larger sac,—pulled thus in one direction by its attachment to the thigh-joint, and in another by those to the spinal column.

The plucking sensations and pain had been accordingly felt by the patient especially on any attempt to straighten the thigh and leg of the affected side. The hernia had evidently become strangulated during the effort to pass the fæces above noticed, but though so short a period had elapsed between that occurrence and death, the parts involved had become so swollen that it was necessary to mutilate them to reduce the constriction.—*L'Experience.*

Sir Astley Cooper has recorded remarkable examples of both mesenteric and mesocolic hernia; but we are disposed to assent to the opinion of our French contemporaries that the above case, which they have named one of *hernia of the broad ligament*, is *sui generis*. It is obvious that in such a case surgical aid is of no avail.—*London Lancet.*

CASE OF TOTAL ABSENCE OF THE SPLEEN.

At Vienna in 1838, the body of an infant was examined which had lived only three days, plunged during the whole of that period in incessant sleep. It had consequently never sought the breast; the meconium and urine had, however, been passed. Only a rudiment existed of the left forearm and hand, and the flesh of the other extremities had a want of tone and firmness. The abdomen and thorax wers tumefied; but in most other respects the child presented little that was unusual in appearance, and the female genital organs were perfectly developed. But on opening the cavities the brain was found softened, and, together with its envelopes, gorged with blood, and a little fluid was present in the ventricles. The lungs were also gorged with blood, and a frothy secretion at their base; but the thoracic organs had a natural developement. The abnormal appearances were mostly in the abdomen. Here the liver was found to extend almost as far into the left as into the right hypochondrium; and to its anterior and inferior border the transverse colon was attached to its entire length. The liver was attached also to the mesocolon, but less intimately. The small intestine held its ordinary position. There was no stomach, unless a small sac, thirteen lines in length by about one-third as wide, and situated at the angle where the œsophagus joined the duodenum, could be so called. There was a pancreas about sixteen lines in length, by four and a half in breadth; but *the spleen was entirely wanting*, and the large intestine had neither *sacculi* nor *appendices epiploicæ*. For further particulars the reader is referred to "Muller's Archiv.," &c., for 1842, p. 59.

We have before had occasion to advert to the fallacies liable to attend the deductions drawn from experiments performed on *live* animals: and the above case affords a suitable opportunity for again remarking that facts furnished by comparative anatomy, together with accurate observations on the defective performance of the animal functions in monsters, are the most legitimate sources whence to derive our conclusions upon points in physiology at present either dark or disputed.—*Ibid.*

BLOOD CORPUSCLES AND SPERMATOZOA OF THE CAMELIDÆ.

After M. Mandle had discovered the elliptical form of the blood discs of the dromedary (*camelus dromedarius*) and paco (*auchenia paco*,) Mr. Gulliver found the same form* in the blood discs of the vicugna (*auchenia vicugna*) and llama (*auchenia glama.*) To complete the history of the blood discs of the camelidæ, it only remained therefore, to examine the red particles of the camel, (*camelus Bactrianus;*) and the following measurements of the blood discs of this animal are extracted from a paper by Mr. Gulliver, recently published in the Proceedings of the Zoological Society of London, part x, page 191:—

| Long Diameter. | Short Diameter. |
|---|---|
| 1-3555 | 1-6000 |
| 1-3000 | 1-8000 |
| 1-4000 | 1-4572 |
| 1-3123 | 1-5876 |

Hence the blood corpuscles of the Bactrian camel, like those of its congeners, are oval; and this form, we believe has not yet been found in the corpuscles of any other mammiferous animal. The circumstance is the more remarkable, since Mr. Gulliver's former observations* have shown that the blood discs of the marsupial mammalia have the circular figure common to the red particles of mammalia generally.

The above measurements are given in vulgar fractions of an English inch; and the numbers beneath the lines are averages estimated from the preceding observations.

*Spermatozoa of the Camel.*—At the meeting of the Zoological Society, April 11th, 1843, Mr. Gulliver read a description of the spermatozoa of the camel, agreeing with that which he formerly gave of the spermatozoa of the dromedary (Proc. Zool. Society, part x, 1842, p. 101)—viz., that they are of the same type as the corresponding animalcules of other mammalia. Although, therefore, there is a singular correspondence of form between the blood discs of the camelidæ and the blood discs of the oviparous vertebrate animals, there is no such resemblance in the spermatozoa.—*Prov. Med. Journ.*

SPERMATOZOA IN THE FLUID OF HYDROCELE.

In a communication made at the last meeting of the Medico-Chirurgical Society, by Mr. Lloyd, of St. Bartholomew's Hospital, the author stated that, in two cases of common hydrocele in which he microscopically examined the fluid withdrawn by tapping, he found numerous spermatozoa. The first case occurred in the early part of last winter. Subsequently to that, in the course of a few weeks, he examined the fluid in four other cases, but did not find any amalcules in them. Three months ago the second case occurred, the patient was sixty-three years of age, and had been previously operated upon for hydrocele about fifteen times. Sixteen ounces of a greenish-yellow fluid, so albuminous as to be quite adhesive, were drawn off. The author counted forty of these microscopical beings in one drop of the fluid. Some of the animalcules were observed to retain their power of motion for three hours after the fluid had been withdrawn. Blood globules, transparent cysts, and small granules, with portions of epithelium, or what much resembled it, were likewise found in the fluid. The authors had lately examined the fluid in other cases of hydrocele, but had not met with spermatozoa in any of them. *Ibid.*

* See Appendix to Gerber's Anatomy, p. 10 and 43.

*See Dub. Med. Press, Nov. 27, 1839; and Wagner's Physiology, by Dr. Willis, part II, p. 234, *note.*

